# Memoranda for Young Practitioners in Midwifery

**Published:** 1837-04

**Authors:** 


ART. XIX.—*Memoranda for Young Practitioners in Midwifery.* By EDWARD RIGBY, M.D., Lecturer on Midwifery at St. Thomas's Hospital, &c.—*London*, 1837. 48mo. pp. 48.

THIS, we think, is the smallest medical book we ever saw: it is certainly the most minute that has come under our critical revision; and we must say that its value, in our opinion, is in the inverse ratio of its bulk. The author informs us, in his preface, that "he has been for some years in the habit of writing on a large tablet a brief outline of each lecture, for the purpose of directing the attention of his class to those facts and general laws connected with obstetric practice which he deemed of peculiar value, and which he was anxious they should not forget." The little volume before us contains these memoranda, which, from their importance, soundness, and clearness, not only merit the attention of every young accoucheur, but ought to be his daily remembrancer in practice.

